# Hydrogels derived from galactoglucomannan hemicellulose with inorganic contaminant removal properties

**DOI:** 10.1039/d1ra06278f

**Published:** 2021-11-08

**Authors:** Leonidas Encina, Elizabeth Elgueta, Bernabé L. Rivas, Miguel Pereira, Felipe Sanhueza

**Affiliations:** Polymer Department, Faculty of Chemistry, University of Concepción Casilla 160-C Concepción Chile brivas@udec.cl; Centro de Investigación de Polímeros Avanzados, CIPA Avenida Collao 1202, Edificio de Laboratorios Concepción Chile e.elgueta@cipachile.cl; Departmento de Ingeniería Química, Facultad de Ingeniería, Universidad de Concepción Casilla 160-C Concepción Chile; Instituto de Materiales y Procesos Termomecánicos, Facultad de Ingeniería, Universidad Austral de Chile Valdivia Chile

## Abstract

The adsorption of Cu(ii), Cd(ii), and Pb(ii) ions onto hydrogels derived from modified galactoglucomannan (GGM) hemicellulose was studied. GGM hemicellulose was modified with methacrylate groups (GGM-MA) to incorporate vinyl groups into the polymeric structure, which reacted later with synthetic monomers such as 2-acrylamido-2-methyl-1-propanesulfonic acid (AMPS). The results show that all the synthesized hydrogels were capable of adsorbing contaminating ions with high adsorption efficiency during short periods of time. Furthermore, an increase in the content of GGM-MA generated a hydrogel (H3) with a similar ion adsorption property to the other hydrogels but with a lesser degree of swelling. The H3 hydrogel had an adsorption capacity of 60.0 mg g^−1^ Cd(ii), 78.9 mg g^−1^ Cu(ii), and 174.9 mg g^−1^ Pb(ii) at 25 °C. This result shows that modified GGM hemicelluloses can be employed as renewable adsorbents to remove Cu(ii), Cd(ii), and Pb(ii) ions from aqueous solutions.

## Abstract 2

The adsorption of Cu(ii), Cd(ii), and Pb(ii) ions onto hydrogels derived from modified galactoglucomannan (GGM) hemicellulose was studied.

## Graphical abstract

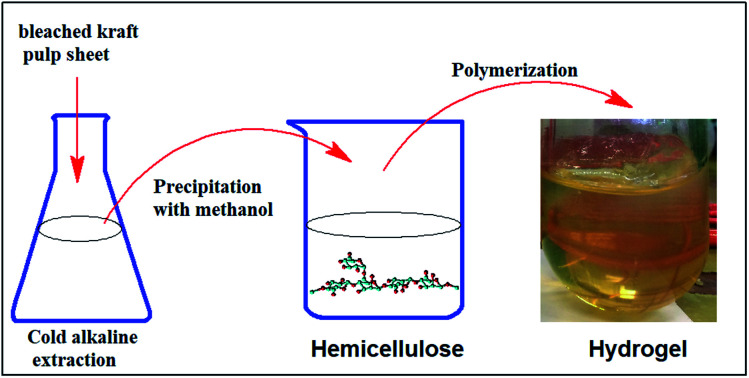

## 1. Introduction

Water is an essential resource for all living beings;^1^ unfortunately, this resource is increasingly scarce due to the lack of rainfall or due to its contamination, currently it is estimated that 1 in 3 people globally do not have access to safe drinking water.^2^ Pollution by toxic metals is a global threat that has accelerated dramatically since the beginning of the industrial revolution. Some of the sources of heavy metal contamination include industries such as mining, pigments, paint coatings, tanneries, electronics, metal plating, power plants (thermal and nuclear), municipal landfills, combustion of fossil fuels and contamination by sewage-sludge agronomic practices.^3,4^ Heavy metal ions can compromise the integrity of various ecological cycles and negatively impact the health of humans through drinking water and the food chain, due to deterioration in water quality. Heavy metal ions such as cadmium, barium, copper, nickel and chromium have been found in alarming concentrations, which can cause acute and chronic diseases in humans and other animals.^5–7^ For example, cadmium pollution has become one of the most serious environmental problems worldwide, and the excessive ingestion of copper brings about serious toxicological concerns, such as vomiting, convulsions, or even death. Lead can cause damage to the central nervous system, kidneys, liver and reproductive system, as well as to basic cellular processes and brain functions.^8–10^ Therefore, eradicating these metal pollutants from water is of the utmost importance.^11^

Various technologies have been applied to the treatment of water contaminated with heavy metals. These technologies include chemical precipitation, ion exchange, flotation, membrane filtration, carbon adsorption, coprecipitation/adsorption, electrochemistry, and reverse osmosis.^12–15^ Nevertheless, these techniques have several inherent disadvantages, such as high costs and the formation of toxic sludge and other waste products that require disposal.^16,17^ In the last decade, numerous studies have been carried out to produce more efficient, environmentally friendly (biobased), and reusable materials for the removal of heavy metals from wastewater. Materials such as lignin,^18^ cellulose,^19^ chitin/chitosan,^20^ hemicellulose,^21^ agricultural waste,^22^ starch^23^ and bark^24^ have been tested with respect to their ability to adsorb metal ions.

Many types of lignocellulosic biomass show effective binding of toxic heavy metals from industrial and environmental effluents. Bioadsorption is an emerging option compared to the conventional methods for the removal of heavy metals, some of which show even higher removal efficiencies than those using conventional methods. Raw material used for bioadsorption is typically low-cost and easily available, and includes agricultural waste or forest residues such as sawdust, bark, or needles.^25,26^ The sorption of metals by wood-based biomass has a large potential in removing metals from aqueous solutions and for their recovery for further use.^27–30^ Among these, cellulose and hemicelluloses have a high potential due to their abundance and to the numerous refining factories that supply and commercialize them, *e.g.*, pulp mills.

Hemicelluloses are heteropolysaccharides composed of different sugar units. They are low molecular-weight biopolymers with a degree of polymerization from 80 to 200. Due to their branched polymeric structure and low molecular weight, their industrial applications are limited.^31–33^ Therefore, their chemical modification provides materials with different properties and uses, allowing the generation of products of greater value and utility.^25,34^ The chemical modification of hemicellulose through the insertion of carboxyl, sulfonic, amide groups, *etc.*, favours its hydrophilic nature and its ability to adsorb polluting ions.^34–37^

The aim of this study is to develop bioadsorbents derived from hemicellulose galactoglucomannan that are capable of adsorbing copper(ii), cadmium(ii), and lead(ii) ions under different experimental conditions. Specifically, previously extracted galactoglucomannan (GGM) hemicellulose is chemically modified by inserting methacrylate groups to incorporate vinyl groups into the polymeric structure that are useful for reacting with synthetic monomers, such as 2-acrylamido-2-methyl-1-propanesulfonic acid (AMPS) to obtain hydrogels with different adsorption capacities for contaminating ions.

## 2. Experimental section

### 2.1 Materials

All chemical reagents were of analytical grade and used as received without further purification. These reagents included 4-(dimethylamino)pyridine (DMAP, 99%, Sigma-Aldrich, Chile), glycidyl methacrylate (GMC, 97%, Sigma-Aldrich, Chile), dimethyl sulfoxide (DMSO, 99%, Sigma-Aldrich, Chile), 2-acrylamido-2-methyl-1-propanesulfonic acid (AMPS, 99%, Sigma-Aldrich, Chile), *N*,*N*-dimethylformamide (DMF, 99%, Sigma-Aldrich, Chile), benzoyl peroxide (BP, 98%, Sigma-Aldrich, Chile), *N*,*N*,*N*′,*N*′-tetramethylethylene-diamine (TEMED, 99%, Sigma-Aldrich, Chile), Cd(NO_3_)·4H_2_O (98%, Merck Chile), Cu(NO_3_)·3H_2_O (99%, Merck Chile), Pb(NO_3_)_2_ (99%, Merck Chile), NaOH (99%, Merck Chile), HNO_3_ (70%, Merck Chile), KOH (90%, Merck Chile), ethanol absolute (Merck Chile), methanol (Technical grade, Diprolab), and HCl fuming (37%, Merck Chile).

### 2.2 Extraction of hemicelluloses

The raw materials and reagents used in the extraction and precipitation of hemicellulose galactoglucomannan included a bleached kraft pulp sheet, potassium hydroxide 5%, hydrochloric acid 37%, technical methanol and distilled water. The bleached kraft pulp was kindly supplied by CMPC, Santa Fe, Chile.

Hemicelluloses were obtained by a selective extraction method that involved cold alkaline extraction and subsequent precipitation with methanol. The bleached kraft pulp sheet was hydrated for 4 h, stirred for 30 min, centrifuged, and pelletized. The pelletized holocellulose had a humidity of 53%. This was added to an Erlenmeyer flask containing a 5% KOH solution and was stirred. Subsequently, it was vacuum filtered, and a 37% HCl solution and methanol were added to the filtrate and left to rest for 16 h to favour the precipitation of the GGM hemicelluloses. Precipitated GGMs were concentrated, centrifuged, and freeze dried.^38–40^

### 2.3 Preparation of the bioadsorbents

The previously extracted hemicelluloses were dissolved in dimethylsulfoxide (DMSO) at 95 °C under constant agitation for 1 h in a round bottom flask immersed in a thermostatic bath. Subsequently, it was allowed to cool to room temperature, and then the catalyst 4-(dimethylamino)pyridine (DMAP) was added under constant stirring. Finally, glycidyl methacrylate (GMA) was added while stirring the reaction mixture constantly for 72 h at 40 °C. At the end of the reaction time, the mixture was cooled and then added dropwise to a 95% ethanol solution while stirring. Subsequently, the solid was left to decant and then vacuum filtered, frozen, and lyophilized.

The synthesis of the hydrogels was carried out in a polymerization flask, to which GGM-MA, solvent (DMF), AMPS monomer, BP initiator and TEMED as a catalyst were added, and stirring was performed for 12 h at 60 °C. After the reaction was finished, the gel formed was left in abundant deionized water for 24 h, filtered under vacuum and dried in a lyophilizer. The synthesis of the hydrogels was carried out in proportions of 10% (H1 hydrogel), 20% (H2 hydrogel) and 40% (H3 hydrogel) in percentage m m^−1^ of the GGM-MA macromonomer with respect to the AMPS, DMF as a solvent, benzoyl peroxide as an initiator and TEMED as a catalyst (10 mol% with respect to the AMPS). Table 1 summarizes the quantities that were used.

**Table 1:** Reagents and quantities used in the synthesis of the hydrogels

Reagents	H1	H2	H3
GGM-MA (g)	0.3 (10%)	0.3 (20%)	0.3 (40%)
AMPS (g)	2.7 (90%)	1.2 (80%)	0.5 (60%)
Benzoyl peroxide (g)	0.32	0.14	0.05
TEMED (mL)	0.19	0.09	0.03
DMF (mL)	10	10	10

### 2.4 Characterization methods

To determine the content of carbohydrates and residual lignin in the pulp, acid hydrolysis was carried out in two stages. First, the pulp sample was hydrolysed with 72% H_2_SO_4_ for 1 h in a water bath at 30 °C. Subsequently, the acid was diluted to a final concentration of 4%, and in a second stage, the mixture was autoclaved at 121 °C for 1 hour. The soluble lignin content was measured in a UV-VIS spectrophotometer (GENESYS 10) at a wavelength of 205 nm. Carbohydrate analysis was performed on a high efficiency liquid chromatograph (HPLC, AMINEX 87P), refractive index detector (YL 9170, IR), 4-channel pump (YL 9110), oven (YL 9131), autosampler (YL 9150), degasser (TL 9101), Aminex column (HPX-87H, pH 1–3) and pre-column cation H. The operating conditions were as follows: flow 0.6 mL min^−1^, 35 °C temperature, 5 mM sulfuric acid as the mobile phase, 20 μL injection volume, and 950–1000 psi working pressure.

The average molecular weight of GGM was determined by size exclusion chromatography, where three ultrahydrogel columns with porosities of 120, 250 and 500 kDa were used, linked in series with an IR detector. The analyses were carried out at 35 °C using an alkaline solution (pH = 13) composed of sodium hydroxide (0.1 mol L^−1^)/sodium acetate (0.2 mol L^−1^) as the eluent with a flow of 0.5 mL min^−1^. Dry samples of isolated xylan were dissolved in the alkaline solution until a concentration of 1 g L^−1^ was obtained. All samples were filtered with a 0.45 μm filter before being injected. The sample injection volume was 20 μL. The columns were calibrated using pullulans as standards.

FT-IR spectra were recorded by a Nicolet Magna 550 spectrophotometer using KBr discs in the spectral range from 400 to 4000 cm^−1^. Scanning electron microscopy (SEM) with X-ray microanalysis was used to analyse the superficial morphology of the hydrogels, and the images were recorded using a JEOL microscope (JSH 6380LV model). The elementary and chemical analyses of the samples were performed by using energy dispersive X-ray spectroscopy (EDX) using an Oxford Instruments INCAx-sight.

The ^1^H and ^13^C NMR spectra were recorded on a Bruker Avance 400 MHz spectrometer, with tetramethylsilane as an internal standard and DMSO-d_6_ as the solvent. Multidimensional ^13^C solid-state magic angle spinning (MAS) NMR was used to identify the chemical interactions present in the H3 hydrogel. Thermogravimetric analysis (TGA) was performed on a NETZSCH 209 F1 Iris model (TA Instruments), and differential scanning calorimetry analysis (DSC) was performed on a NETZSCH 204 F1 Phoenix at a heating rate of 10 °C min^−1^. The pH measurements were conducted using a Mettler Toledo Instrument pH metre, Seven Compact pH/ion model. All cation measurements were performed on a PerkinElmer PinAAcle 900F atomic absorption spectrometer (AAS) using an electrode discharge lamp (EDL) and a flame atomizer with 2.5 L min^−1^ of acetylene and 10 L min^−1^ of air. The detected wavelengths were 228.8 nm for cadmium, 283.3 nm for lead, and 324.7 nm for copper.

### 2.5 Swelling behaviour

The swelling study was carried out by adding 100 mg of hydrogel and 100 mL of deionized water to a beaker. It was left to stand for 24 h at room temperature, and then it was filtered by gravity in a glass crucible and the percentage of swelling was determined by using the difference between the dry weight (*W*_d_) and swollen weight (*W*_s_) of the hydrogel, according to eqn (1).1
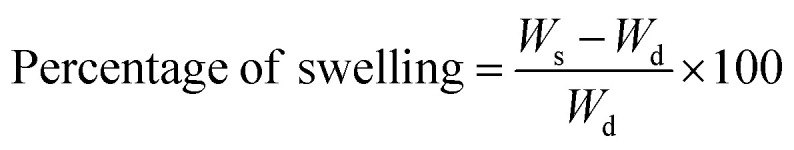


### 2.6 Sorption experiments

The adsorption of Cu(ii), Cd(ii), and Pb(ii) ions onto the hydrogels was analysed using a batch equilibrium procedure. Studies of the influence of pH on adsorption were assessed at pH 3, 4, and 5. The experiments were carried out with 20 mg of dry hydrogel in 5 mL of 100 mg L^−1^ metal ion solution for 60 min at 140 rpm and 25 °C. Then, the sample was centrifuged, and the heavy metal ion content in the supernatant was measured by atomic absorption spectroscopy (AAS). Adsorption studies as a function of contact time between the hydrogel and heavy metal solution were assessed at 10, 20, 30, 40, 60, 90, 120, 240, 360, and 1440 min at pH 4, 25 °C, and 140 rpm, using 20 mg of hydrogel and 5 mL of metal ion solution of 100 mg L^−1^ Cu(ii), Cd(ii), and Pb(ii) ions. At the end of the contact time, the samples were centrifuged and the supernatants were measured by AAS.

The sorption capacity at different concentrations was determined at pH 4 for 60 min at 140 rpm and 25 °C using initial concentrations of 100, 300, 500, 700, and 1300 mg L^−1^ Cd(ii) and 100, 300, 500, 700, 1000, and 1500 mg L^−1^ Cu(ii) and Pb(ii) ions. For this experiment, 20 mg of hydrogel was placed in contact with 5 mL of each cationic solution, then the samples were centrifuged and the supernatant was measured by AAS. The adsorption capacity at equilibrium was determined by using eqn (2), where *q*_e_ is the amount of metal ions adsorbed per weight of hydrogel at equilibrium (mg g^−1^), *C*_0_ is the initial concentration, *C*_e_ is the final concentration (mg L^−1^), *V* is the volume of the cation solution (L), and *W* is the weight of the dried hydrogel (g).2
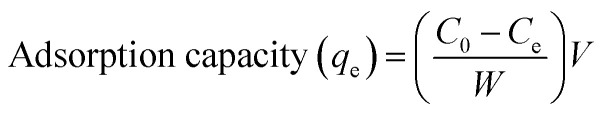


Selectivity studies were performed at pH 4, 140 rpm, 25 °C, and 60 min of contact using 20 mg of hydrogel and a multicomponent solution containing Cu(ii), Cd(ii), and Pb(ii) ions at a concentration of 100 mg L^−1^ for each metal. Then, the mixtures were centrifuged and the supernatants were measured by AAS.

The selectivity coefficient was determined by using eqn (3), where *q*_e1_ and *q*_e2_ are the adsorption capacities of the adsorbent (mg g^−1^) by the metal ions M1 and M2, respectively.3
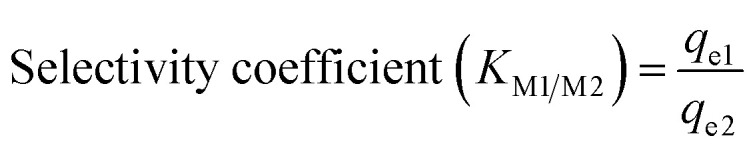


Desorption studies were performed using 20 mg of dry hydrogel loaded with Cu(ii), Cd(ii), or Pb(ii) ions under optimal conditions. The dried hydrogel charged with the respective metal ion was placed in contact with 5 mL EDTA solution of concentration 0.1 mol L^−1^ at 25 °C, 140 rpm and at an optimal pH and time for each metal ion. Subsequently, the sample was centrifuged and the amount of desorbed metal ions was measured by AAS. All experiments were carried out in duplicate.

The distribution of metal ions onto the hydrogels can be described by Langmuir and Freundlich isotherm models. The experiments were performed using 20 mg of hydrogel and solutions with concentrations ranging from 100 to 1300 mg L^−1^ for Cd(ii) and 100 to 1500 mg L^−1^ for Cu(ii) and Pb(ii) at 25, 35, and 45 °C for 24 h at pH 4 and 140 rpm. Adsorption kinetic studies were analysed using the pseudo-second-order equation, and the experimental data were obtained from the adsorption study as a function of contact time between the hydrogel and cationic solution.

According to the previous results, a column equilibrium study was carried out to analyse the adsorption of Pb(ii) on the hydrogels in a continuous process. Accordingly, a glass column (1.2 × 11 cm) with a frit (pore size 100–160 μm), Tygon hoses and peristaltic pump was used. The experiments were carried out by packing the column with 52.6 mg of hydrogel, which corresponded to 0.652 mL of hydrated hydrogel in the column that reached a bed height of 0.6 cm and a Pb(ii) solution of 20 mg L^−1^ at pH 4 at a constant flow rate of 1.38 mL min^−1^. The experimental data were adjusted to the Thomas model.^41,42^

## 3. Results and discussion

### 3.1 Synthesis and characterization of the bioadsorbent materials

**Fig. 1:**
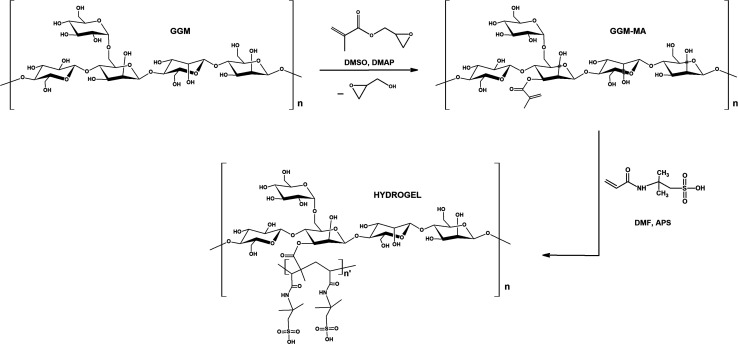
Hydrogel synthesis scheme.

GGM hemicellulose was obtained by cold alkaline extraction of a kraft pulp sample of *Pinus radiata*, which was mainly composed of glucose (88.0%), mannose (9.9%), arabinose (1.5%), and lignin (0.5%). The average molecular weight was 35 kDa. Subsequently, this GGM hemicellulose generated the macromonomer GGM-MA by reaction with DMAP, which then generated the hydrogels under study by reaction with different proportions of AMPS. Fig. 1 shows a reaction scheme.

**Fig. 2:**
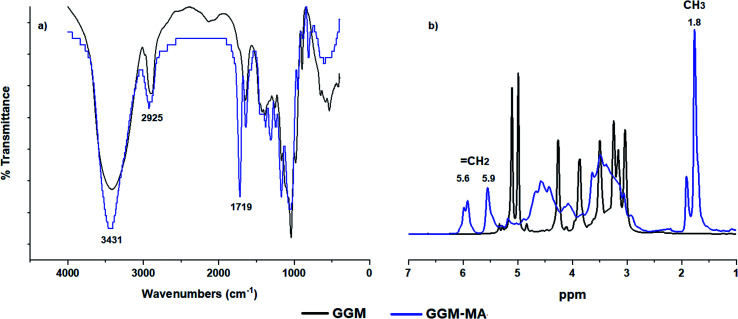
FT-IR spectrum (a) and ^1^H NMR spectra (b) of GGM and GGM-MA.

Infrared spectroscopic studies were performed to determine the presence of the functional groups in galactoglucomannan (GGM) hemicellulose, macromonomers with methacrylate groups (GGM-MA), and hydrogels. The FT-IR spectra obtained for GGM (black line) and GGM-MA (blue line) are shown in Fig. 2a. In the GGM-MA spectrum, the presence of an absorption band at 1719 cm^−1^ is associated with the carboxyl groups (C=O) of the methacrylate groups inserted in the hemicellulose. Furthermore, the presence of absorption bands at 2925 cm^−1^ and 3431 cm^−1^ corresponds to C–H and hydroxyl groups, respectively.

Nuclear magnetic resonance studies of ^1^H, ^13^C and DEPT 135 were performed in deuterated dimethyl sulfoxide (DMSO-d_6_) as the solvent. Through these studies, it was possible to determine the structures of GGM hemicellulose and the GGM-MA macromonomer. These studies also allowed us to confirm the transesterification reaction between GGM hemicellulose and glycidyl methacrylate, where methacrylic acid groups were inserted into the hemicellulose structure to generate a GGM-MA macromonomer, releasing a glycidol group (see Fig. 1).

**Fig. 3:**
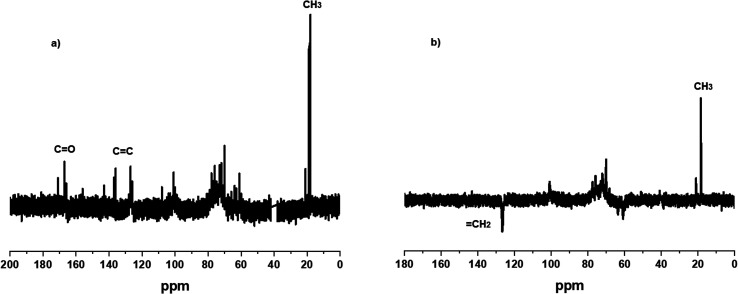
^13^C (a) and DEPT 135 (b) spectra for the GGM-MA macromonomer.

Fig. 2b shows the ^1^H NMR spectra of GGM (black line) and GGM-MA (blue line). In the GGM-MA spectrum (blue line), the appearance of two signals at 5.6 and 5.9 ppm, corresponding to vinyl hydrogens (C=CH_2_), and at 1.8 ppm, a signal attributed to methyl hydrogens (CH_3_) of the inserted methacrylate groups, was observed. Fig. 3a shows the ^13^C and DEPT 135 spectra for the GGM-MA macromonomer. The ^13^C NMR spectrum shows signals at 18 ppm corresponding to the methyl groups (CH_3_), signals at 126 and 136 ppm corresponding to the vinyl carbons of the methacrylate group (C=C), and the signal at 166 ppm corresponding to the carboxylic carbon. To better appreciate the signals, the septet centred at 39.52 ppm corresponding to the DMSO-d_6_ solvent was eliminated from the graph. For studies by DEPT 135, positive signals (upward signals) are indicative of CH and CH_3_ groups, while negative signals (downward signals) are indicative of CH_2_ groups and quaternary carbons have no signals. Fig. 3b shows the presence of the CH_3_ group (positive signal) of the methacrylate group inserted at 18 ppm, and at 126 ppm, the negative signal corresponds to the CH_2_ group of the vinyl group.

**Fig. 4:**
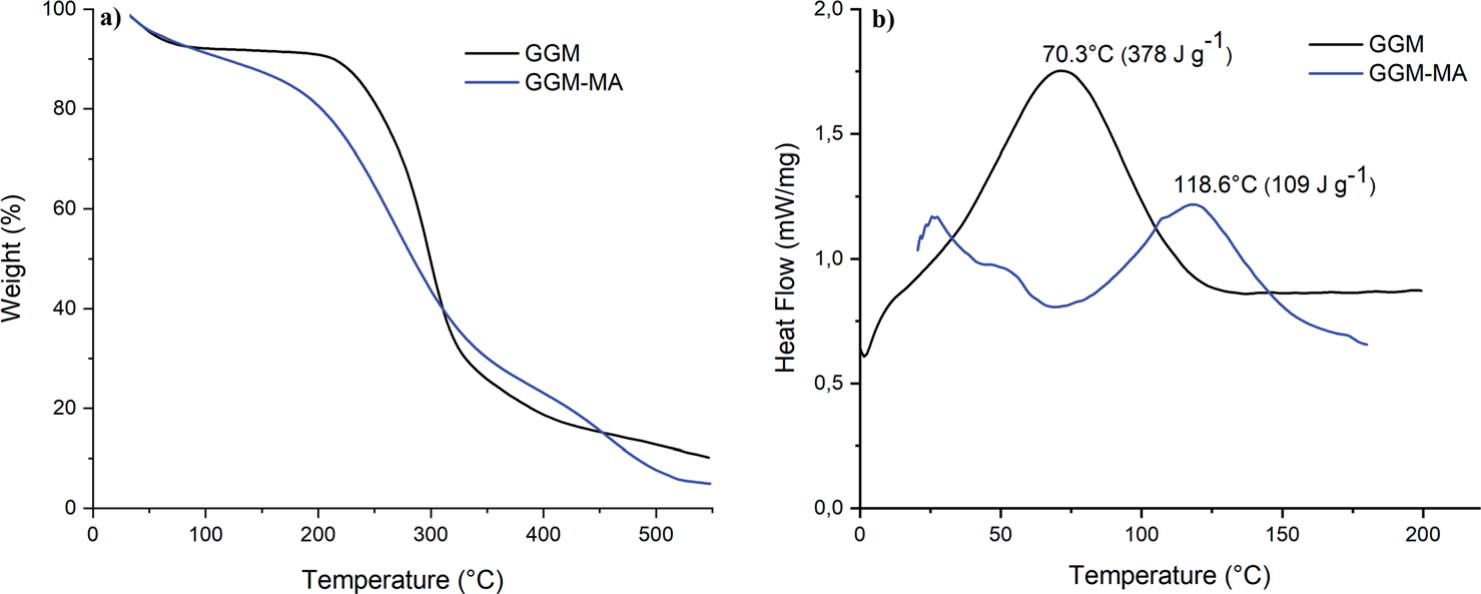
Thermal properties. Thermogravimetric analysis (TGA) (a) and differential scanning calorimetry (DSC) (b) of GGM and GGM-MA.

Thermal properties were characterized by thermogravimetric analysis (TGA, see Fig. 4a) and differential scanning calorimetry (DSC, see Fig. 4b) at a heating rate of 10 °C min^−1^. GGM hemicellulose exhibited 50% decomposition at 299 °C, and at 546 °C, it had 90% decomposition. In addition, it had a melting temperature of 70.3 °C with an enthalpy of 378 J g^−1^. The GGM-MA macromonomer underwent 50% decomposition at 283 °C, while 90% decomposition was obtained at 480 °C. GGM-MA had a melting temperature of 118.6 °C and an enthalpy of 109 J g^−1^.

**Fig. 5:**
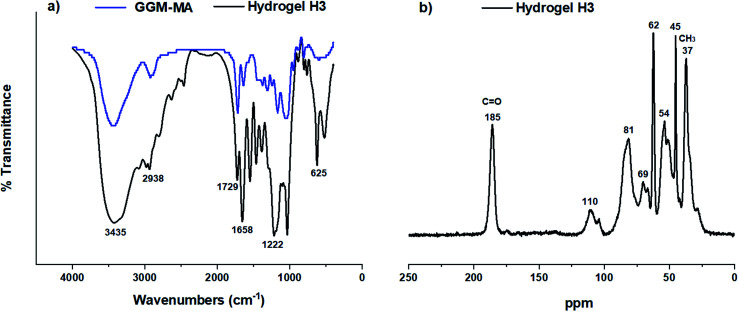
FT-IR spectrum (a) and ^13^C MAS solid-state NMR (b) spectra for the H3 hydrogel.

Fig. 5a shows the FT-IR spectrum of the H3 hydrogel, where bands are observed at 625 cm^−1^ and 1222 cm^−1^ corresponding to the vibrations of the –SO– and –SO_3_H groups of the AMPS inserts, respectively. This emphasizes that sulfonic groups favour the adsorption of polluting ions. The absorption bands at 1658 cm^−1^ and 1729 cm^−1^ are associated with the vibrations of the C=O group of the amide and the ester, respectively. The absorption bands between 2900 and 3000 cm^−1^ correspond to the C–H vibrational bands (Csp^2^ and Csp^3^), and the absorption band at 3435 cm^−1^ is associated with the vibration of the hydroxyl groups (–OH). Using ^13^C MAS solid-state NMR, it was possible to identify the chemical shift present in the H3 hydrogel. Fig. 5b shows the ^13^C MAS solid-state NMR spectra for the H3 hydrogel. The chemical shifts at 37, 45 and 54 ppm are assigned to the AMPS-derived carbons, while the chemical shifts at 62, 69, 81 and 110 ppm are assigned to the galactoglucomannan fraction. The signal at 185 ppm corresponds to the C=O group.

**Fig. 6:**
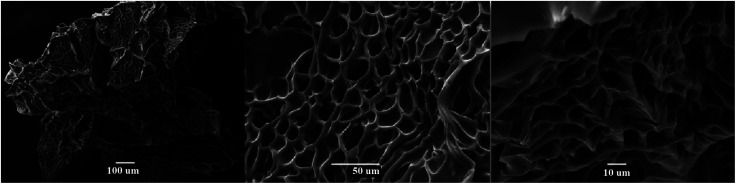
SEM images of the H3 hydrogel.

It is important to know the surface characteristics of a material that is used in adsorption processes. Through SEM studies, it was possible to observe that the synthesized hydrogels had a highly porous surface morphology, which favours interaction with contaminating ions. Fig. 6 shows micrographs obtained for the H3 hydrogel at different magnifications, where a high surface porosity with interconnected pores can be observed.

### 3.2 Swelling behaviour

The degree of swelling represents the water absorption capacity per gram of hydrogel, which is a very important property and provides valuable information on how the ions can diffuse through the pores of the cross-linked matrix and then be retained by the functional groups present in the polymer matrix. This property is influenced by various structural factors of the polymeric matrix, such as the degree of cross-linking, concentration, nature of the functional groups (p*K*), degree of ionization, and hydrophilicity. The degree of swelling of the hydrogels allows us to know the maximum water absorption capacity, which is directly related to the presence of the hydrophilic functional groups in the polymeric matrix that contributes to the difference in osmotic pressure with the solution; therefore, the water absorption compensates for the difference generated.^43,44^

The hydrogels H1, H2 and H3 obtained in proportions of 10, 20 and 40% m m^−1^ of GGM-MA, respectively, have sulfonic functional groups, which have a high hydration capacity and a low p*K* (p*K*_a_ = 1.9) and are dissociated in a wide range of pH values, which favours the difference in osmotic pressure and therefore favours the absorption of water. The results obtained indicate that H1 was capable of absorbing 35.1 g H_2_O per g hydrogel, H2 31.1 g H_2_O per g hydrogel and H3 22.7 g H_2_O per g hydrogel.

### 3.3 Effect of pH on adsorption

The pH of the aqueous solution is an important parameter in the ion adsorption process, and the effect of pH variation on the adsorption of Cu(ii), Cd(ii), and Pb(ii) ions was analysed at pH 3, 4, and 5. The range of pH was restricted to avoid the precipitation of metals, because the formation of Cd(OH)_2_ begins at pH 7.0, of Cu(OH)_2_ at pH 5.6, and of Pb(OH)_2_ at pH 7.8.^12^

The results are summarized in Table 1. They show that the three hydrogels had a high adsorption capacity for Cu(ii), Cd(ii), and Pb(ii) ions, having a retention percentage that varied between 90% and 98% when using an initial solution of 100 mg L^−1^. Furthermore, as the percentage composition of GGM in the hydrogel increased, the adsorption capacities were maintained.

Table 2 shows that the adsorption of Cu(ii) ions was favoured at pH 5 for the three hydrogels; in turn, the adsorption of Cd(ii) and Pb(ii) ions for the three hydrogels was favoured at pH 4. Furthermore, the sum of the retention was higher at pH 4; therefore, pH 4 is defined as the optimal working pH. At high pH values, the amount of hydrogen ions in solution was lower; therefore, the competition between metal ions and protons decreased, favouring the adsorption of cations on the active sites of the hydrogels. However, at higher pH values, it was also possible to find precipitates of Cu(ii), Cd(ii), and Pb(ii) ions as hydroxides; therefore, the disappearance of the metal ions from the solution could have been due to a combined process of adsorption/precipitation. In contrast, at a low pH, the quantity of hydrogenic ions in solution was greater, generating competition and repulsion of charges, making it difficult for the adsorption of cations in the hydrogels, which decreased.

**Table 2:** Adsorption study as a function of pH

Hydrogel	pH	Metal ions (mg g^−1^)		
		Cu(ii)	Cd(ii)	Pb(ii)
H1	3	22.9	19.5	26.2
	4	23.0	24.6	28.2
	5	32.3	14.7	25.4
H2	3	24.1	21.0	28.0
	4	23.5	27.0	29.4
	5	32.0	15.0	26.4
H3	3	23.0	20.3	26.6
	4	23.6	26.9	27.8
	5	31.4	15.7	25.5

### 3.4 Effect of contact time on the adsorption of metal ions

Through the pH studies, it was found that the H3 hydrogel, which had the highest percentage of GGM, had a high cation adsorption capacity (higher than 95%); therefore, the following studies were based on this hydrogel.

**Fig. 7:**
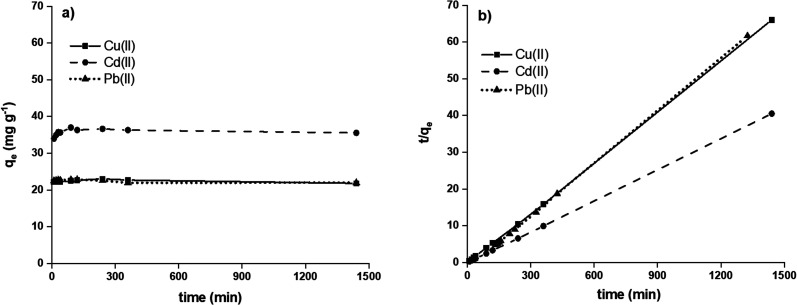
Effect of contact time on the adsorption of metal ions (a) and adsorption kinetics (b).

Adsorption studies as a function of contact time between the hydrogel and heavy metal ion solution were assessed at 10, 20, 30, 40, 60, 90, 120, 240, 360, and 1440 min at a pH 4 for the H3 hydrogel. The results are shown in Fig. 7a. The H3 hydrogel can adsorb the cations Cu(ii), Cd(ii), and Pb(ii) during short periods of contact, having an almost constant adsorption through time. Thus, after 40 min of contact, 97% Cu(ii) ions (22.2 mg g^−1^), 94% Cd(ii) ions (34.9 mg g^−1^), and 99% Pb(ii) ions (22.6 mg g^−1^) were adsorbed using the initial solutions with concentrations of Cu(ii) = 95.4 mg L^−1^, Cd(ii) = 151 mg L^−1^, and Pb(ii) = 92.7 mg L^−1^. This adsorption of cations during short periods of contact can be attributed to the high affinity of the sulfonic acid/sulfonate groups for cationic species due to an ion exchange process.^45,46^ Through this study, 40 min was chosen as the optimal contact time.

### 3.5 Adsorption kinetics

The experimental data obtained from the adsorption studies as a function of contact time were analysed under the pseudo-second-order kinetic model (see eqn (4)), obtaining a good experimental fit for the three divalent cations under study (see Fig. 7b). This equation has been successfully applied to the adsorption of metal ions, herbicides, dyes, organic substances, and oils from aqueous solutions.^47^4
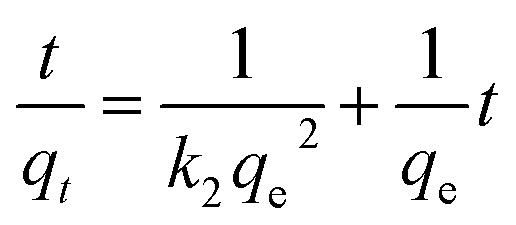
where *q*_*t*_ and *q*_e_ are the adsorption capacity of metal ions (mg g^−1^) at time *t* and equilibrium, respectively, and *k*_2_ (g mg^−1^ min^−1^) is the velocity constant for pseudo-second-order adsorption. The correlation factor (*R*^2^, see Table 3) indicates that the limiting stage was adsorption rather than mass transfer and the removal mechanism was chemisorption. The kinetic parameters are given in Table 3, where it is seen that the adsorption rate constant *h* (mg g^−1^ min^−1^) has the following trend, Cd(ii) > Pb(ii) > Cu(ii).

**Table 3:** Kinetic parameters for H3 hydrogel

Cation	*R* ^2^	*K*_2_ (g mg^−1^ min^−1^)	*q*_e_ (mg g^−1^)	*h* (mg g^−1^ min^−1^)
Cu(ii)	0.99	0.010	21.7	5.1
Pb(ii)	1.00	0.021	21.9	10.3
Cd(ii)	1.00	0.012	35.5	15.5

### 3.6 Adsorption–desorption studies

Through adsorption–desorption studies, it is possible to know the regeneration capacity of the hydrogel. The desorption of the cations was evaluated using a 0.1 mol L^−1^ EDTA solution, and the results are given in Table 4, where it can be seen that EDTA was able to efficiently elute the three cations under study.

**Table 4:** Adsorption–desorption studies

Cation	Adsorption^a^ (%)	Adsorption^a^ (mg g^−1^)	Desorption (%)
Cu(ii)	99.7	33.0	97.8
Cd(ii)	99.8	29.6	91.8
Pb(ii)	92.2	27.3	100

a 40 min of contact; concentration: 135.2, 123.5, and 123.3 mg L^−1^ of Cu(ii), Cd(ii), and Pb(ii), respectively at pH 4.

### 3.7 Maximum adsorption capacity

The maximum adsorption capacity was determined by the number of functional groups and their chemical nature. This study was performed at different initial concentrations (100–1500 mg L^−1^) for Cu(ii), Cd(ii), and Pb(ii) ions at a pH 4.

**Fig. 8:**
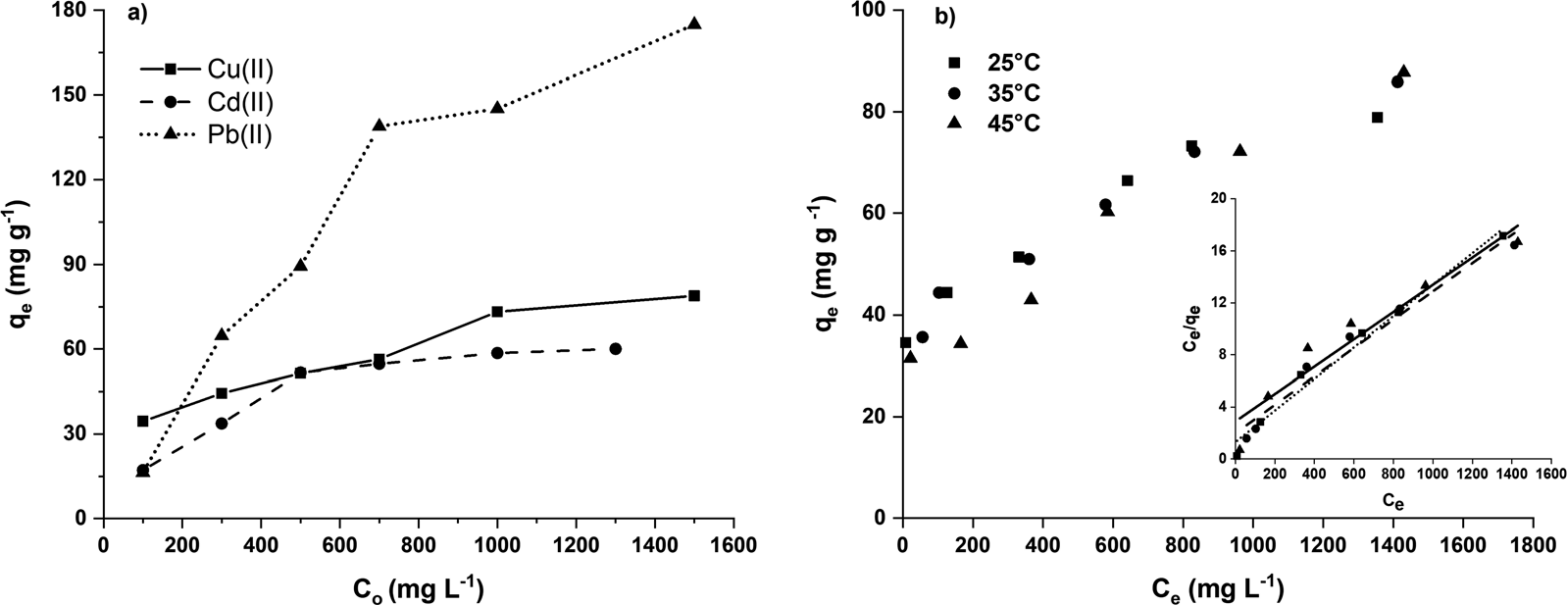
Maximum adsorption capacity (a) and adsorption isotherms (b).

The results are shown in Fig. 8a, where it can be seen that for the Cu(ii) and Cd(ii) ions, plateaus were reached at initial concentrations of 1500 and 1300 mg L^−1^, respectively. Under these experimental conditions, an adsorption capacity of 78.9 mg g^−1^ for Cu(ii) and 60.0 mg g^−1^ for Cd(ii) was achieved. The adsorption of Pb(ii) ions was 174.9 mg g^−1^, and a plateau or saturation value was not reached. The adsorption order follows the trend, Pb(ii) > Cu(ii) > Cd(ii). The adsorption capacity of the H3 hydrogel for different heavy metals is mainly due to the difference in the binding ability of functional groups to heavy metal ions. The presence of functional groups –OH, CONH_2_ and –SO_3_H^48^ in hydrogel networks enables high adsorption of Pb(ii) ions, this is probably due to the high ionic radius of the Pb(ii) ion (1.19 Å) compared to Cd(ii) (0.97 Å) and Cu(ii) (0.69 Å) ions.^49^

Furthermore, the Fig. 8a shows that the increase in the initial concentration led to an increase in the amount adsorbed per gram of bioadsorbent. This behaviour may be due to the fact that this greater quantity of Pb(ii) ions can provide a higher driving force for effective collision between ions and active sites of the hydrogel, thereby promoting the transfer of ions between the two phases (diffusion from the solution phase to hydrogel phase) and more collisions between ions and active sites of the hydrogel (Chen *et al.* 2020;^50^ Wang *et al.* 2021^51^). This behaviour can be attributed to an increase in driving force, given by the concentration gradient, which exceeds the resistance of the mass transfer between the bioadsorbent and the adsorption medium due to the increase in the diffusion rate of the metal ions.

### 3.8 Adsorption isotherms and thermodynamic parameters

The distribution of metal ions between the liquid and solid phases in an adsorption process in equilibrium can be described by Langmuir (eqn (5)) and Freundlich (eqn (6)) isotherm models.^52^5
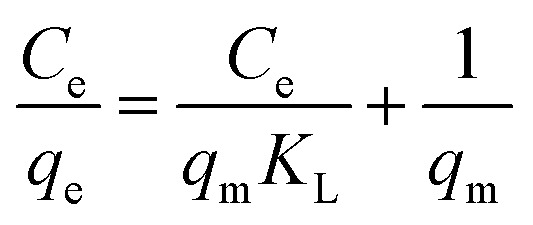
6
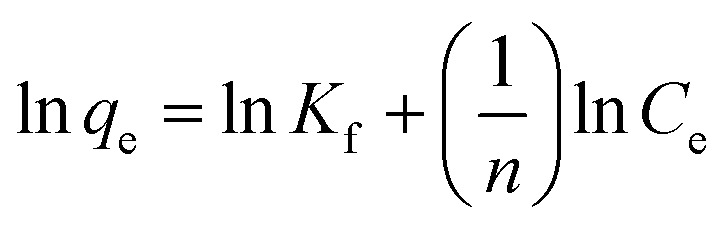
where *q*_e_ is the amount adsorbed at equilibrium (mg g^−1^), *C*_e_ is the equilibrium concentration (mg L^−1^), and *q*_m_ is the maximum amount of adsorbate that the adsorbent can adsorb for the formation of a monolayer (mg g^−1^). The parameter *q*_m_ is used in evaluating adsorption performance, especially in cases where the adsorbent does not reach full saturation, because it allows an indirect comparison among different adsorbents. *K*_L_ is the Langmuir constant related to the affinity of active sites (L mg^−1^). *K*_f_ is the Freundlich constant associated with the efficiency of adsorption, and *n* is a dimensionless parameter that indicates the favourability of adsorption.

The Langmuir constant allows us to calculate the *R*_L_ parameter that indicates how favoured the adsorption is, where *R*_L_ > 1 is unfavourable, *R*_L_ = 0 is irreversible, *R*_L_ = 1 is linear, and 0 < *R*_L_ < 1 is favourable (see eqn (7)).7
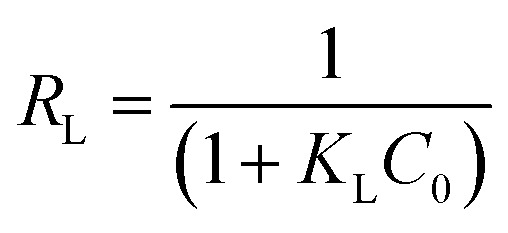


Table 5 shows the results obtained for the adsorption isotherms and thermodynamic parameters. These results indicate that the Langmuir isotherm has a higher correlation with the experimental data than the Freundlich isotherm (see Table 5). Fig. 8b shows the adsorption isotherms for Cu(ii) ions at 25, 35 and 45 °C, and the Langmuir isotherms are presented inside the graph. Using the Langmuir parameters, the *R*_L_ values were between 0 and 1, which indicates that the adsorption process was favourable. It is also observed that the *q*_m_ values decreased with increasing temperature, which may be due to the increase in the mobility of the ions adsorbed on the surface, which may favour desorption. The values of *q*_m_ have a trend of Pb(ii) > Cu(ii) > Cd(ii).

**Table 5:** Langmuir parameters for cation sorption onto H3 hydrogel

	Cation	25 °C	35 °C	45 °C
**Langmuir**				
*q*_m_	Cu(ii)	82.6	72.4	69.4
	Cd(ii)	58.8	60.0	46.7
	Pb(ii)	151.5	143.0	92.6
*K*_L_	Cu(ii)	0.001	0.009	0.010
	Cd(ii)	0.071	0.067	0.072
	Pb(ii)	0.018	0.013	0.014
*R* ^2^	Cu(ii)	0.96	0.97	0.97
	Cd(ii)	1.00	1.00	1.00
	Pb(ii)	0.92	0.97	0.92
*R*_L_	Cu(ii)	0.40	0.34	0.44
	Cd(ii)	0.20	0.17	0.50
	Pb(ii)	0.80	0.40	0.50

**Thermodynamic parameters**				
ln *K*_0_	Cu(ii)	4.34	1.95	0.87
	Cd(ii)	5.10	3.63	3.45
	Pb(ii)	3.91	2.21	1.83
Δ*G*° (kJ mol^−1^)	Cu(ii)	−10.77	−5.00	−2.31
	Cd(ii)	−12.64	−9.30	−9.13
	Pb(ii)	−9.70	−5.67	−4.85
Δ*H*° (kJ mol^−1^)	Cu(ii)	−137.43		
	Cd(ii)	−65.46		
	Pb(ii)	−82.50		
Δ*S*° (kJ mol^−1^ K^−1^)	Cu(ii)	−0.42		
	Cd(ii)	−0.18		
	Pb(ii)	−0.25		

Using the experimental results at different temperatures, it is possible to calculate the thermodynamic parameters associated with the adsorption process of the cations Cu(ii), Cd(ii) and Pb(ii). The parameter values for entropy (Δ*S*°) and enthalpy (Δ*H*°) can be obtained by means of the linear form of the van't Hoff equation (eqn (8)) and the Gibbs free energy (Δ*G*°), which informs us if the adsorption process was spontaneous, and can be determined by eqn (9).8
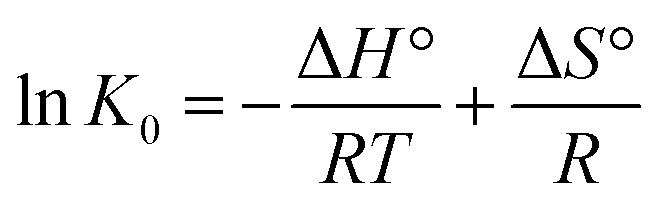

9 Δ*G°* = −*RT* ln *K*_0_

where *K*_0_ is the apparent equilibrium constant, and their values were determined from the intercept from plotting ln(*C*_ads_/*C*_e_) *versus C*_ads_, where *C*_ads_ is calculated as *C*_0_–*C*_e_ (*C*_ads_ is the equilibrium concentration of adsorbate on the adsorbent and *C*_0_ is the initial concentration (mg L^−1^)). The values of *K*_0_ are summarized in Table 5.

The values for enthalpy (Δ*H*°) and entropy (Δ*S*°) were determined from the slope and intercept of the plot of ln *K*_0_*versus T*^−1^, and the results are given in Table 5. The enthalpy values provide information on the type of adsorption and, through the results obtained, we can estimate that the adsorption process was exothermic chemical sorption. The negative Gibbs free energy indicates that the adsorption was spontaneous for the three metals in the studies; therefore, it is concluded that the adsorption process under working conditions is viable.

The Freundlich parameters are summarized in Table 6, where *n*, a parameter that indicates the intensity of the adsorption, has values between 1 and 10, which indicates that the adsorption was favourable.

**Table 6:** Freundlich parameters for cation sorption onto H3 hydrogel

Freundlich	Cation	25 °C	35 °C	45 °C
*n*	Cu(ii)	6.20	4.52	5.26
	Cd(ii)	4.19	3.13	3.82
	Pb(ii)	2.87	2.46	2.40
*K*_f_	Cu(ii)	22.80	14.20	15.60
	Cd(ii)	16.60	9.60	11.01
	Pb(ii)	18.37	10.49	7.70
*R* ^2^	Cu(ii)	0.92	0.87	0.81
	Cd(ii)	0.92	0.87	0.81
	Pb(ii)	0.92	0.87	0.81

Table 7 shows the results obtained for different hemicellulose-derived adsorbents, in which Peng *et al.* and Wu *et al.*^35,53^ reported adsorption values higher than those obtained in our study. However, Ayoub *et al.* and Lian *et al.*^54,55^ reported adsorption capacities lower than ours. The adsorbents obtained by Peng, Ayoub, Wu and Lian *et al.* used unmodified hemicellulose, while our hydrogels were obtained through the chemical modification of galactoglucomannan hemicellulose by inserting methacrylate groups, which were subsequently polymerized with AMPS. The novelty in this work lies in the use of environmentally friendly, biodegradable, non-toxic waste biomaterials that have the ability to interact-adsorb contaminating ions, such as Cd(ii), Cu(ii) and Pb(ii). This capacity be enhanced by the insertion of different functional groups capable of trapping ions, in order to obtain bioadsorbents with high adsorption capacities of species harmful to society and the environment.

**Table 7:** Cu(ii), Cd(ii) and Pb(ii) adsorption capacities obtained for different hemicellulose-derived adsorbents

Type of hydrogels (adsorbent)	Cu(ii) (mg g^−1^)	Cd(ii) (mg g^−1^)	Pb(ii) (mg g^−1^)
Xylan-rich hemicelluloses-based hydrogel (Peng *et al.*^35^ 2012)	—	464	810
Hemicellulose–chitosan biosorbent for heavy metal removal (Ayoub *et al.*,^54^ 2013)	0.95	—	2.9
Hemicelluloses–chitosan with TiO_2_ (Wu *et al.*^53^ 2017)	158.7	78.1	—
Hemicelluloses-based hydrogel and its application as an adsorbent towards heavy metal ions (Lian *et al.*^55^ 2018)			5.54
Bioadsorbents derived from galactoglucomannan hemicellulose with inorganic contaminants removal properties (this study)	82.6	58.8	151.5

### 3.9 Selectivity studies

Selectivity studies were performed for the H3 hydrogel with a multicomponent solution containing Cu(ii), Cd(ii), and Pb(ii) ions at concentrations of 135 mg L^−1^, 123 mg L^−1^, and 124 mg L^−1^, respectively. The results show that the H3 hydrogel was not selective because it adsorbed the three divalent cations with adsorption capacities greater than 90% with high efficiency, retaining 10.79 mg g^−1^ Cu(ii), 9.89 mg g^−1^ Cd(ii) and 9.27 mg g^−1^ Pb(ii). Using eqn (3), it was possible to determine the selectivity coefficient, where *K*_Cu/Pb_ and *K*_Cu/Cd_ were 1.16 and 1.09, respectively.

### 3.10 Column studies

**Fig. 9:**
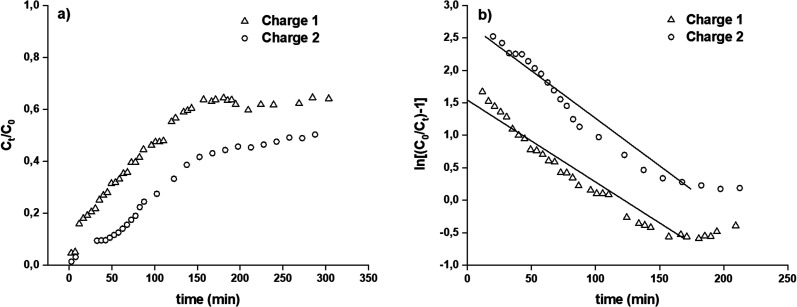
Column studies, breakthrough curve for two charges (a) and graphical fit to the Thomas model (b).

The experiments were carried out by packing the column with 52.6 mg of hydrogel, which corresponded to 0.652 mL of hydrated hydrogel in the column that reached a bed height of 0.6 cm and a Pb(ii) solution of 20 mg L^−1^ at a pH 4 and a constant flow rate of 1.38 mL min^−1^. To elute, a 0.1 mol L^−1^ of EDTA solution was used. The experimental data were adjusted to the Thomas model. For the column studies, two contact or charge (Ch), and elution or discharge (Dch) cycles were carried out using the working conditions described in Table 7. Fig. 9a displays the breakthrough curve for the two charges. Column adsorption properties were analysed using 52.6 mg of the H3 hydrogel equivalent to a bed volume of 0.652 mL, a Pb(ii) solution of 20 mg L^−1^ at a pH 4 and a flow rate of 1.38 mL min^−1^. To elute, a 0.1 mol L^−1^ of EDTA solution was used.

The adsorption capacity of the column was calculated using eqn (10) and (11), and the efficiency of the column was calculated using eqn (12).10
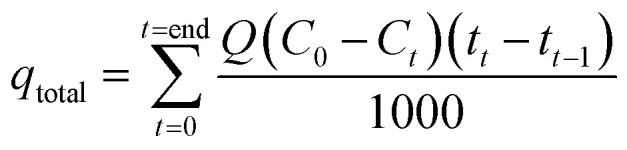
11
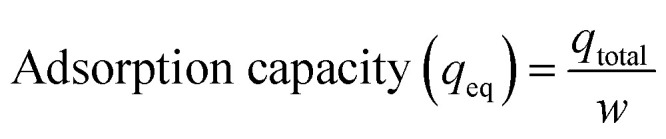
12
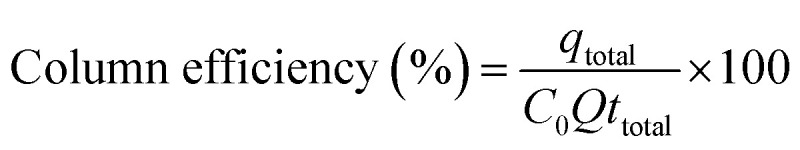
where *q*_total_ is the total amount of the adsorbed cation (mg), *q*_eq_ is the total amount of the adsorbed cation per amount of H3 hydrogel (mg g^−1^), *C*_0_ and *C*_*t*_ are the concentrations (mg L^−1^) at initial and time *t*, respectively, *w* is the mass of the adsorbent used in the column (g), and *Q* is the operational flow (mL min^−1^). The results are given in Table 7, where column adsorption results are lower than those obtained by batch because the residence times (*t*_R_) of the contaminating solution with the solid in the column were significantly low (28.4 s), which limited the contact of the cation with the hydrogel. To obtain a high removal of the pollutant from the aqueous medium, the *t*_R_ should be as high as possible.

The Thomas model is used to predict the adsorption curve in the fixed-bed column under different operational conditions. The linear form of the Thomas model is described in eqn (10) and Fig. 9b shows the graphical fit to the Thomas model.13
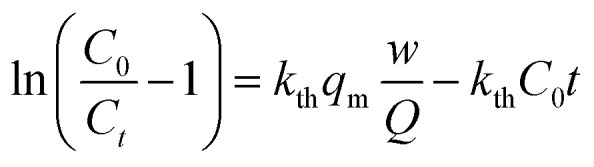
where *C*_0_ and *C*_*t*_ are the concentrations (mg L^−1^) of the inlet and outlet at time *t*, respectively. *K*_th_ is the Thomas adsorption rate constant (mL h^−1^ mg^−1^), *q*_m_ is the column maximum equilibrium capacity of the adsorbent (mg g^−1^), *w* is the mass of the adsorbent used in the column (g), *Q* is the operational flow (mL min^−1^) and *t* is the flow time (min). The values of *q*_m_ and *K*_th_ were obtained from the interception and slope, respectively, of the plot of ln[(*C*_0_/*C*_*t*_) − 1] *versus t*. The results are summarized in Table 8 and show a good fit with the Thomas model, indicating that the adsorption process was controlled by the mass transfer between the solid and liquid interfaces.

**Table 8:** Operational parameters of Pb(ii) adsorption–desorption onto H3 hydrogel in the fixed-bed column^a^

Operation condition					Thomas constant			Adsorption column parameters	
Cycle	*C*_0_	*Q*	Total volume fed	Total time	*K*_th_	*q*_m_	*R* ^2^	Adsorption capacity (*q*_e_)	Column efficiency (%)
Ch 1	23.0	1.38	409.2	305	0.52	95.4	0.960	69.3	38
Dch 1	0.1*	1.38	187.0	135					80
Ch 2	19.7	1.38	405.0	288	0.82	84.1	0.941	73.9	50
Dch 2	0.1*	1.38	180.0	130					52

a The units of *C*_0_ (mg L^−1^), * (mol L^−1^), *Q* (mL min^−1^), total volume fed (mL), total time (min), *K*_th_ 10^−3^ (mL min^−1^ mg^−1^), *q*_m_ (mg g^−1^), adsorption capacity (mg g^−1^). Charge (Ch) with cations and discharge (Dch) with EDTA.

## 4. Conclusions

The present work shows the feasibility of using a high-performance biobased and modified hemicellulose material as an inorganic contaminant adsorbent by means of a two-step chemical modification. The results show that the bioadsorbents or hydrogels are capable of adsorbing copper(ii), cadmium(ii), and lead(ii) ions. Studies based on the contact time show that the adsorption of Cu(ii), Cd(ii) and Pb(ii) cations occurred very quickly, with high adsorption during short periods of contact, which favours their industrial use. Through adsorption–desorption studies, it is concluded that EDTA was a good eluent, allowing the adsorption properties of the H3 hydrogel to be maintained in the different cycles.

The experimental data show a good fit with the Langmuir isotherm, and the thermodynamic values indicate that the process was exothermic and spontaneous. The high value of enthalpy indicates that the determining process was chemical sorption.

By column or continuous method, it was observed that the H3 hydrogel, when in contact with a solution of Pb(ii) ions at a pH 4, did not undergo great alterations in its loading capacity, which shows promising results for its reuse. Furthermore, the Thomas model shows a good fit with the experimental data.

## Funding

FONDECYT (grant no. 11170753 and no. 1190469).

## Ethical approval

The authors declare the research did not involve either human participation or animal testing.

## Author contributions

The manuscript was written with contributions of all authors. All authors have given approval to the final version of the manuscript.

## Conflicts of interest

The authors declare that they have no conflict of interest.

## Supplementary Material
